# Preparation, Pharmacokinetics, Biodistribution, Antitumor Efficacy and Safety of Lx2-32c-Containing Liposome

**DOI:** 10.1371/journal.pone.0114688

**Published:** 2014-12-15

**Authors:** Hongbo Wang, Jianqiao Zhang, Guangyao Lv, Jinbo Ma, Pengkai Ma, Guangying Du, Zongliang Wang, Jingwei Tian, Weishuo Fang, Fenghua Fu

**Affiliations:** 1 Key Laboratory of Molecular Pharmacology and Drug Evaluation (Ministry of Education of China), School of Pharmacy, Yantai University, Yantai, China; 2 State Key Laboratory of Bioactive Substances and Functions of Natural Medicines, Institute of Materia Medica, Chinese Academy of Medical Sciences and Peking Union Medical College, Beijing, China; 3 Department of clinical medicine, Binzhou Medical College, Yantai, China; 4 State Key Laboratory of Long-acting and Targeting Drug Delivery Technologies (Luye Pharma Group Ltd.), Yantai, China; University of South Florida, United States of America

## Abstract

Lx2-32c is a novel taxane that has been demonstrated to have robust antitumor activity against different types of tumors including several paclitaxel-resistant neoplasms. Since the delivery vehicles for taxane, which include cremophor EL, are all associated with severe toxic effects, liposome-based Lx2-32c has been developed. In the present study, the pharmacokinetics, biodistribution, antitumor efficacy and safety characteristics of liposome-based Lx2-32c were explored and compared with those of cremophor-based Lx2-32c. The results showed that liposome-based Lx2-32c displayed similar antitumor effects to cremophor-based Lx2-32c, but with significantly lower bone marrow toxicity and cardiotoxicity, especially with regard to the low ratio of hypersensitivity reaction. In comparing these two delivery modalities, targeting was superior using the Lx2-32c liposome formulation; it achieved significantly higher uptake in tumor than in bone marrow and heart. Our data thus suggested that the Lx2-32c liposome was a novel alternative formulation with comparable antitumor efficacy and a superior safety profiles to cremophor-based Lx2-32c, which might be related to the improved pharmacokinetic and biodistribution characteristics. In conclusion, the Lx2-32c liposome could be a promising alternative formulation for further development.

## Introduction

Paclitaxel has been widely used as a chemotherapeutic agent in the treatment of a broad range of cancers [1]–[3]. However, its clinical usefulness has been limited by drug-resistance and delivery vehicle-related toxic effects, especially regarding the hypersensitivity induced by cremophor EL castor oil [4]–[6]. Therefore, novel taxanes involving cremophor EL free formulations that retain their sensitive regarding paclitaxel-resistant cancers would be of great clinical benefit.

Previously, we reported for the first time on Lx2-32c, a novel taxane semisynthesized from cephalomannine [7], [8]. This compound displayed robust anticancer activity against several cancer cell lines both *in vitro* and *in vivo*, especially against several paclitaxel-resistant lines such as A549/taxol and A2780/paclitaxel [8], [9]. Based on this finding, Lx2-32c has been viewed as a potential candidate to overcome paclitaxel-resistance in the clinic. However, because of its aqueous insolubility, Lx2-32c must be dissolved in the same vehicle (cremophor EL and anhydrous ethanol [1∶1 V/V]) as the paclitaxel, which will introduce similar severe side effects to those of taxol [4], [10]. Therefore, an improved formulation, which could eliminate the adverse effects associated with the solvent while retaining similar anticancer activity, would greatly benefit cancer patients and accelerate the development process.

To overcome the solvent challenge regarding taxane, some cremophor-free or reduced cremophor EL paclitaxel formulations, such as liposomes, Genexol- PM, AI850, Genetaxyl or an albumin-bound nanoparticle formulation of paclitaxel, have been approved or developed in clinical trials [11]–[17]. Amongst them, liposomes have been used to encapsulate a variety of pharmacological agents, such as doxorubicin and paclitaxel (Lipusu, paclitaxel liposome for injection) [18], [19]. It has been well documented that liposome encapsulation of doxorubicin could reduce local irritation and vesicant action without a significant decrease in antitumor effect [20]. In addition, paclitaxel when encapsulated in a liposome (Lipusu) instead of a conventional excipient has been shown to have a markedly reduced toxicity while retaining equal efficacy in mice and rat cancer models [18]. Consequently, a liposome-based formulation of Lx2-32c might be effective in cancer treatment without the toxic side effects induced by cremophor-based Lx2-32c. In the present study, the Lx2-32c liposome was prepared and its antitumor effect, toxicity, biodistribution and pharmacokinetics were evaluated and compared with those of cremophor-based Lx2-32c.

## Methods

### Chemicals and animals

Lx2-32c was obtained from the Chinese Academy of Medical Sciences, and the purity of the compound used in the present study was higher than 98% as checked by HPLC. The 100% ethanol and cremophor EL castor oil were kindly supplied by the Beijing Union Pharmaceutical Factory. Lecithin was provided by Avanti Polar Lipids, Inc. Cholesterol was purchased from the Hubei Kang-baotai Fine-Chemicals Co. Ltd.

Male C57BL/6J mice (18–22 g) were provided by the Beijing HFK Bioscience Co., Ltd, and male SD rats (200–240 g) were obtained from the Shandong Luye Pharmaceutical Co., Ltd. The animals were housed in a light and temperature-controlled room (21–22°C; humidity 60–65%) and maintained on a standard diet and water. All of the experimental protocols were approved by Committee on the Ethics of Animal Experiments of Yantai University. All surgery was performed under sodium pentobarbital anesthesia, and all efforts were made to minimize suffering.

### Preparation of Lx2-32c liposomes

Lx2-32c liposomes were prepared using a film dispersion method followed by a lyophilization technique. Briefly, Lx2-32c (60 mg), lecithin (720 mg) and cholesterol (108 mg) were dissolved in chloroform. After 5 min of stirring, the organic solvent was evaporated on a rotary evaporator under reduced pressure at 40°C to obtain a membrane. The resulting membrane was dissolved by the addition of PBS (pH = 7.4) to obtain the Lx2-32c liposome solution. The solution obtained using this process was disrupted in a 200-W ultrasonic homogenizer for 20 min. Then, the remaining solution was lyophilized to dried Lx2-32c liposomes using a freeze dryer system (Labconco, USA) and their microstructure was observed using a scanning electron microscope. Particle size was evaluated by means of a particle size analyzer (Mastersizer 2000, Malvern Instruments). The encapsulation efficacy of LX2-32c was measured using high-performance liquid chromatography (HPLC; Shimadzu LC-20A, JPN; C18 column, 250×4.6 mm; 5 µm).

### Evaluation of antitumor effects and toxicity in B16 tumor-bearing C57BL/6J mice

C57BL/6 mice were used to establish xenograft tumors of murine melanoma (B16) as previously reported [21]. In this experiment, the tumors were isolated from donor mice and implanted in the dorsum of recipient mice by means of subcutaneous injection. The day after implantation, the animals were randomized into three groups each containing 10 animals: the control group was given a single dose of 0.9% NaCl by intraperitoneal injection; the Lx2-32c cremophor EL group (the compound) was administrated in three doses by intraperitoneal injection in a 10 ml/kg injection volume (30 mg/kg) twice every week; the Lx2-32c liposome group was administrated in three doses by intraperitoneal injection in a 10 ml/kg injection volume (30 mg/kg) twice every week.

Hypersensitivity was assessed according to the grading standards detailed in Table 1 in line with our previous report [22]. Blood samples were collected in tubes containing EDTA for hematological analysis, and in tubes without anticoagulation agent for the evaluation of clinical chemical parameters after the final dose. The weight of the tumor obtained from each mouse was measured to evaluate the antitumor effects. Hematological parameters such as red blood cell (RBC) count, hemoglobin (HGB) level, white blood cell (WBC) count and platelet (PLT) count were compiled at the 407 Naval Hospital of Yantai, China. For the clinical chemistry study, blood samples were centrifuged at 4000 rpm for 10 min at room temperature without anticoagulation. The level of creatine kinase MB (CK-MB) in serum was also assayed at the 407 Naval Hospital of Yantai.

**Table 1:** Hypersensitivity reactions grading standard.

Grade	Clinical signs
0/−	Normal
1/+	Dyspnea, syncope, gatism
2/++	Disturbance, head shaking
3/+++	Shortness of breath, drowsiness
4/++++	Mortality

### Pharmacokinetic experiments in male SD rats

The pharmacokinetics of the Lx2-32c liposomes and the Lx2-32c cremophor EL were explored using SD rats after a single intraperitoneal injection at a dose of 30 mg/kg. Briefly, around 500 µl of blood from the posterior orbit was collected into a heparinized vacutainer tube before compound administration and at 0.17, 0.25, 0.5, 1, 2, 4, 8, 12, 24 and 48 h post administration. The blood samples were centrifuged at 8000 rpm for 10 min at 4°C to obtain plasma samples, which were kept frozen at −80°C until analysis. Each plasma sample was treated with 1 ml of acetonitrile and vortexed for 30 s followed by centrifugation at 12000 rpm for 10 min at 4°C. Then, the supernatants were transferred to other test tubes. The Lx2-32c concentration in the plasma samples was quantified using a Diamonsil-C18 (4.6 mm×250 mm; 5 µm) HPLC column at a temperature of 30°C. The mobile phase was 80∶20 (methanol:water) at a flow rate of 1 ml/min. The effluent was detected at 225 nm and the area under the peak was used for quantification. Pharmacokinetic parameters were evaluated using Winnonlin Software (Version 6.1, Pharsight Corporation).

### Biodistribution of Lx2-32c in B16 tumor-bearing C57BL/6J mice

The mice bearing the xenograft tumor model were separated into Lx2-32c liposome and Lx2-32c cremophor EL groups (40/group) for the biodistribution study. Three animals were chosen from each group at 0.17, 0.25, 0.5, 1, 2, 4, 8, 12, 24 and 48 h post intraperitoneal injection of a 30 mg/kg dose. The hearts and tumors were collected, weighed and frozen at −80°C until assayed. Bone marrow was harvested from femurs using a carefully standardized protocol that involved flushing 500 µl of normal saline through the marrow cavity and collecting the effluent for analysis.

All tissue samples were homogenized in 5 ml acetonitrile. The homogenizer was rinsed with 1 ml acetonitrile to recover the residual drug. The samples were placed on ice and centrifuged for 10 min at 12000 rpm. The supernatants were transferred to other test tubes. The concentration of Lx2-32c in all tissue samples was quantified using the same method and conditions as used for the pharmacokinetic experiments.

### Statistical analyses

The results were presented as mean ± SD. Comparisons between more than 2 groups were performed by analysis of variance (one way ANOVA), then Student t test were performed. P<0.05 was used as the level of statistical significance unless indicated otherwise.

## Results

### Characterization and properties of the Lx2-32c liposome

**Figure 1:**
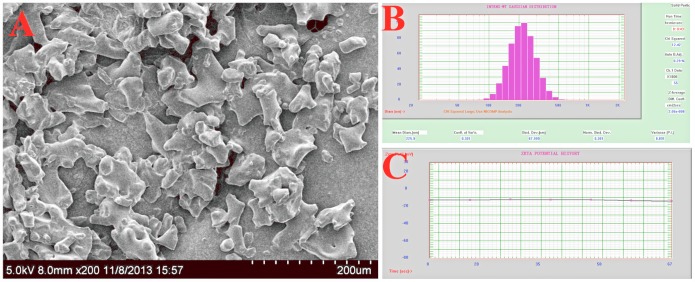
Characterization of the Lx2-32c liposome. A, Scanning electron microscope photograph of Lx2-32c liposome; B, The mean diameter and polydispersity index (PDI) of freshly prepared Lx2-32c liposome; C, The average zeta potential of Lx2-32c liposome.

The Lx2-32c liposomes were tested with a mean diameter of 225.9 nm (Fig. 1B), and the average zeta potential was –14.16 mV (Fig. 1C), in which their appearance under scanning electron microscopy was shown (Fig. 1A), The encapsulation efficiency was about 87% and the loading efficiency was 5.3%as determined by HPLC (S1 and S2 Tables in S1 File). No obvious changes were observed on the mean diameter, polydispersity index and encapsulation efficiency for the freeze-dried liposome powder stored in −20°C for at least 10 months (S3 Table in the S1 File).

### Antitumor effect of Lx2-32c liposome against B16 xenograft tumor in C57BL/6J mice

The antitumor activity was evaluated using xenograft tumor models. The results showed that the Lx2-32c liposome could significantly inhibit the growth of B16 xenograft tumors compared with the control group (Table 2; *P<0.01*); antitumor activity was similar to that in the Lx2-32c cremophor EL group. However, the body weight of animals in the Lx2-32c liposome group was significantly higher relative to that in the Lx2-32c cremophor EL group (Table 2; *P<0.01*).

**Table 2:** Inhibitory effects of Lx2-32c liposome on the xenograft tumor growth of B16 in C57BL/6J mice.

Group	Dosage (mg/kg)	Body weight (g)	Body weight gain (g)	Tumor weight(g)	Inhibition Rate (%)
Control	-	29.44±1.96	4.24±1.53	3.63±0.76	–
Cremophor-based	30	24.00±1.05*	-2.30±1.25*	1.53±1.16*	57.85
liposome	30	27.8±1.99^#^	2.20±1.87* ^, #^	1.66±0.68*	54.27

Data are expressed as means ± SD (n = 10).

*: *p<0.05,* compared with that in control group;^ #^: *p<0.05*, compared with that in Cremophor-based Lx2-32c group.

### Hypersensitivity reactions

The mice were injected intraperitoneally with Lx2-32c in cremophor EL or Lx2-32c liposomes at a dose of 30 mg/kg. Their behavior was subsequently observed and their hypersensitivity reactions were ranked according to the criteria detailed in Table 1. As shown in Table 3, almost all of the animals injected with Lx2-32c cremophor solution were observed to have acute hypersensitivity reactions (grade 3) at 2–5 min post administration; they recovered within 30 min. In contrast, all of the animals in the Lx2-32c liposome group were found to have no or much milder reactions (grade 0 or 1).

**Table 3:** Hypersensitivity grade of Lx2-32c liposome and cremophor-based Lx2-32c in mice.

No.	sterile saline	Cremophor-based Lx2-32c	Lx2-32c liposome
1	–	+++	+
2	–	+++	–
3	–	+++	–
4	–	+++	+
5	–	+++	–
6	–	+++	–

### Hematologic examination

After **2** weeks of administration, the animals in the Lx2-32c cremophor EL group were observed with a markedly reduced WBC count compared with that in the control group (Table 4, *P<0.05*). However, the animals in the Lx2-32c liposome group were found to have a much less pronounced reduction in the WBC count compared with that in the Lx2-32c cremophor group (*P<0.05*). In addition, a significant decrease in the HGB concentration was observed in the animals in the Lx2-32c cremophor group, which was not observed in the Lx2-32c liposome group (Table 4). No significant effect on the RBC and PLT counts was observed in either the Lx2-32c liposome or the Lx2-32c cremophor group (Table 4).

**Table 4:** The effect of Lx2-32c liposome on WBC, RBC, PLT counts, HGB concentration and CK-MB in C57BL/6J mice.

Group	Dosage(mg/kg)	WBC counts (10^9^/L)	RBC counts(10^12^/L)	PLT counts (10^9^/L)	HGB (g/L)	CK-MB (U/L)
Control		17.3±1.65	3.2±0.6	364.1±98.6	64.6±10.9	240.0±25.6
Cremophor-based	30	3.1±1.3*	3.2±1.4	460.6±142.2	49.2±5.3*	409.6±86.7*
liposome	30	10.7±5.9* ^,^ #	3.2±0.8	3764.8±162.4	65.8±8.6#	269.8±33.8#

Data are expressed as means ± SD (n = 10).

*p<0.05, compared with that in control group;

# *p<0.05*, compared with that in Cremophor-based Lx2-32c group.

### Serum myocardial enzyme

CK-MB is a popular biomarker that is used to monitor cardiac injury in the clinic [23]. As shown in Table 4, a marked increase in the activity of CK-MB relative to that in the control group was observed in the animals in the Lx2-32c cremophor group (*P<0.05*); a significant reduction in CK-MB was found in the Lx2-32c liposome group compared with that in the cremophor EL group (*P<0.05*).

### Pharmacokinetic analysis

**Figure 2:**
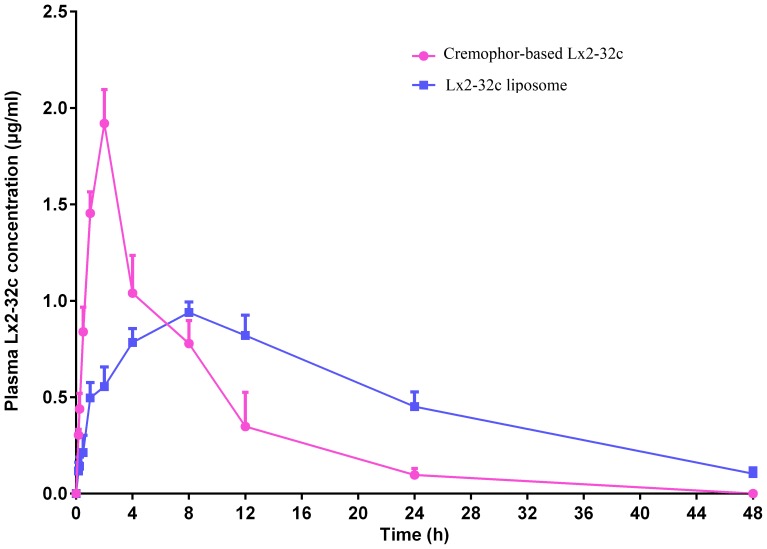
Lx2-32c Plasma concentration–time profile in SD rats following a single i.p. dose of Lx2-32c liposome and cremophor-based Lx2-32c at 30 mg/kg. Data are expressed as mean ± SD (n = 4).

The pharmacokinetic profiles of Lx2-32c in the liposome and cremophor groups were calculated using the non-compartmental analysis with Winnonlin Software and listed as the mean ± SD in Table 5. As shown in Fig. 2, the plasma drug concentration observed was significantly higher in the Lx2-32c liposome group than that in the Lx2-32c cremophor group (*P<0.01*) at 12 h, 24 h and 48 h post administration, and the accumulation and clearance of Lx2-32c occurred over a considerably shorter period in the Lx2-32c cremophor group compared with the Lx2-32c liposome group.

**Table 5:** Mean pharmacokinetic parameters of Lx2-32c liposome and cremophor-based Lx2-32c in SD rats.

Parameter	Cremophor-based Lx2-32c	Lx2-32c liposome
AUC_(0-t)_ (mg/L *h)	15.16±1.41	23.37±2.71
MRT_(0-t)_ (h)	7.94±0.71	19.52±0.77
C_max_ (µg/µl)	1.92±0.63	0.94±0.45
CLz/F (L/h/kg)	2.01±0.72	1.19±0.59
Vz/F (L/kg)	16.63±1.07	20.52±0.91

Data are expressed as mean ± SD (n = 4).

AUC: Area Under Curve; MRT: Mean Retention Time; CLz/F: Clearance; Vz/F: Apparent Volume of Distribution.

### Biodistribution of Lx2-32c

**Figure 3:**
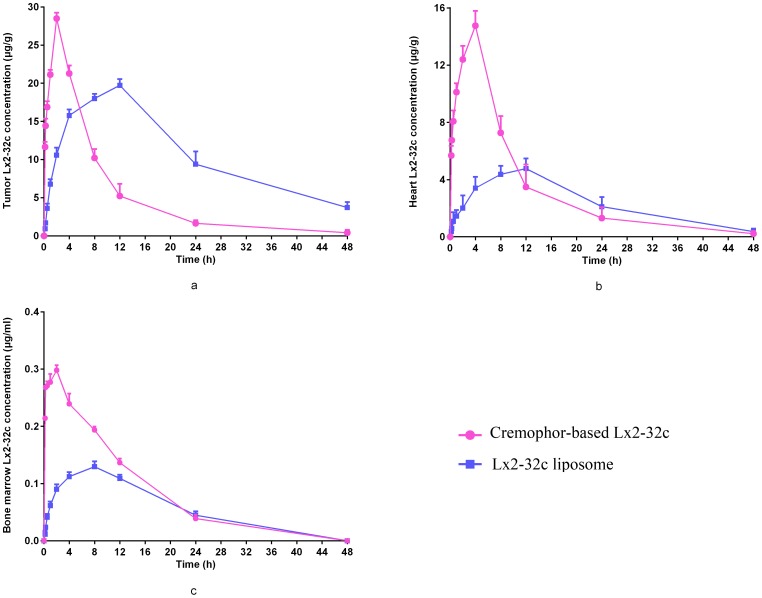
Lx2-32c tissue distribution–time after a single i.p. dose of Lx2-32c liposome and cremophor-based Lx2-32c at 30 mg/kg. (a) tumor; (b) heart; (c) bone marrow. Data are expressed as mean ± SD (n = 4).

**Figure 4:**
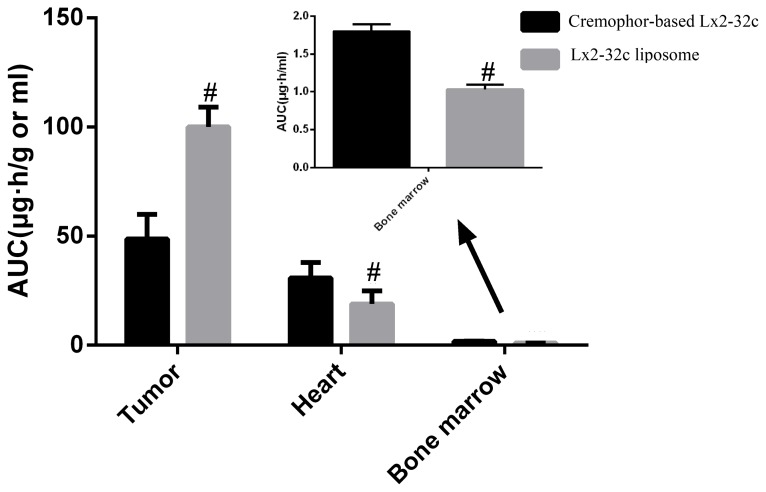
Lx2-32c tissue distribution (AUC) comparative of a single i.p. dose of Lx2-32c liposome and cremophor-based Lx2-32c at 30 mg/kg. Data are expressed as mean ± SD (n = 4). #:*P<0.05*, compared with that in the cremophor-based Lx2-32c group.

The distribution of Lx2-32c was explored after administration, and the results demonstrated that Lx2-32c reached a peak concentration in the tumor at 12 h after liposome injection and at 2 h after Lx2-32c cremophor injection (Fig. 3). The drug concentration in the tumor was significantly lower in the Lx2-32c liposome group than that in the Lx2-32c cremophor group within the first 4 h post administration (*P<0.01*); it was much higher for the Lx2-32c liposomes than for Lx2-32c cremophor at 8, 12, 24 and 48 h post administration (*P<0.01*). However, the distribution of the drug in the heart tissue (Fig. 3b) and bone marrow (Fig. 3c) differed from that in the tumor; drug uptake in these organs was much high in the Lx2-32c cremophor group relative to the Lx2-32c liposome group. The drug uptake was calculated and the data indicated that the AUC_0–48 h_ was approximately 1.5-fold higher for the tumor, while it was about 2.8- and 1.2-fold lower for heart and bone marrow in the Lx2-32c liposome group compared with that in the Lx2-32c cremophor group (Fig. 4).

## Discussion

Lx2-32c is a novel taxane derivative semisynthesized from cephalomannine, which was shown to retain sensitivity to paclitaxel in resistant cancer cells both *in vitro* and *in vivo* [8], [9]. To explore an alternative formulation without cremophor, Lx2-32c liposome was developed and evaluated in the current study. Our data demonstrated clearly that Lx2-32c liposome could produce a similar antitumor effect, milder hypersensitivity reactions, lower bone marrow toxicity and lower cardiotoxicity; in addition, it achieved high drug uptake and changed the biodistribution compared with cremophor-based Lx2-32c, which indicated that the Lx2-32c liposome could be an alternative formulation that merits further development.

Taxane is a class of anticancer drugs with robust antitumor activities against different malignancies in the clinic [23]. However, cremophor, the main ingredient in taxane solvents, is always associated with much severer toxic effects, such as hypersensitivity reactions induced by unwanted complement activation [4]–[6]. Indeed, our data showed that almost all the animals injected with cremophor-based Lx2-32c solution were observed to have different degrees of hypersensitivity reactions; these were much milder or not observed in the Lx2-32c liposome group of animals. Based on our previous study, we estimated that the Lx2-32c liposome did not or only lightly induced complement activation compared with cremophor-based Lx2-32c. Importantly, the antitumor activity of Lx2-32c liposomes was similar to that in the cremophor-based Lx2-32c solution when evaluated in terms of reduction in xeograft tumor weight (Table 2). The following toxic effects, however, were much less severe as assayed using change in body weight, in which the animals in Lx2-32c liposomes were observed with obviously improved values of body weight and body weight gain compared with that in cremophor-based Lx2-32c solution group (Table 2).

Bone marrow suppression and cardiotoxicity are the reported common side effects of taxol, which can easily be detected by means of features such as leukopenia and increased expression of biomarkers of myocardial injury, such as CK-MB [24]. In the current study, decreased leukocyte and neutrocyte counts, and increased CK-MB levels as well as bone marrow hypoplasia, were noted in almost all of the animals treated with cremophor-based Lx2-32c; the magnitude of all of these parameters was remarkably reduced in animals injected with Lx2-32c liposomes. All of these findings indicated that this alternative liposome formulation has a much safer toxicity profile than the cremophor formulation. Similar findings have also been reported for liposome-encapsulated doxorubicin and Lipusu, in which the lower toxic effects might be related to lower drug uptake in the target organs [18], [25].

Free drugs, such as paclitaxel and doxorubicin, can be rapidly cleared by absorption through the peritoneal lining and entry into the systemic circulation; this drug clearance pathway can be changed by encapsulation of drugs in liposomes [26], [27]. As a result, liposome-encapsulated drugs may display sustained high concentrations in the immediate vicinity of the target site, and enhanced tumor uptake of the drug. An example is doxorubicin encapsulated in liposomes, which has been shown to be slowly released into the abdominal cavity from disrupted liposomes in a model of peritoneally disseminated cancer [28]. In the present study, higher drug uptake and longer retention time were observed in the tumor tissues of mice injected with Lx2-32c liposomes relative to that injected with cremophor-based Lx2-32c; at the same time the Lx2-32c liposome group was noted as having a much lower peak drug concentration in rat after i.p. injection. Furthermore, all the animals treated with Lx2-32c liposome exhibited a marked delay before the drug entered the systemic circulation; this was partly manifested by a lower distribution and peak concentrations in some normal tissues such as the heart and bone marrow, and much lower subsequent toxic effects. The possible reason for this is that the lymphatic transit of Lx2-32c liposomes from the peritoneum to the systemic circulation was limited to some degree, when cells induced a severe blockage in the lymphatic drainage from the peritoneum [29].

In conclusion, Lx2-32c liposome was evaluated for the first time *in vitro* and *in vivo.* It displayed equal antitumor efficacy to cremophor-based Lx2-32c but considerably lower toxicity (which was related to its lower uptake in normal tissues), and high drug levels in both the circulation system and tumor tissue. Our results thus showed that the Lx2-32c liposome could be an attractive formulation for further evaluation regarding its use in cancer therapy.

## Supporting Information

S1 File (DOC)
