# Supplementary material for: Preparation, Pharmacokinetics, Biodistribution, Antitumor Efficacy and Safety of Lx2-32c-Containing Liposome
Source: PLoS One. 2014 Dec 15;9(12):e114688. doi: 10.1371/journal.pone.0114688 (PMC4266495; doi:10.1371/journal.pone.0114688)
Supplement: S1 File — (DOC) [file pone.0114688.s001.doc]

**Supplementary Information for: Preparation, pharmacokinetics, biodistribution, antitumor efficacy and safety of Lx2-32c-containing liposome**

Hongbo Wang ^1,#^, Jianqiao Zhang ^1,#^, Guangyao Lv ^1^, Jinbo Ma^3^, Pengkai Ma ^1^, Guangying Du ^1^, Zongliang Wang ^1^, Jingwei Tian ^1,4^, Weishuo Fang ^2^, Fenghua Fu ^1^

1: Key Laboratory of Molecular Pharmacology and Drug Evaluation (Ministry of Education of China), School of Pharmacy, Yantai University, Yantai 264005, China;

2: State Key Laboratory of Bioactive Substances and Functions of Natural Medicines, Institute of Materia Medica, Chinese Academy of Medical Sciences and Peking Union Medical College, Beijing, 100050, PR China.

3: Binzhou Medical College, Yantai 264005, China;

4: State Key Laboratory of Long-acting and Targeting Drug Delivery Technologies (Luye Pharma Group Ltd.), Yantai 264003, China

#: These authors contribute equally to this study.

Correspondence: Dr. Hongbo Wang and Dr. Fenghua Fu, School of Pharmacy, Yantai University, Yantai 264005, P.R.China; Tel: +86 535 6706060; Fax: +86 535 6706066; E-mail:hongbowangyt@gmail.com or [fufenghua@sohu.com](mailto:fufenghua@sohu.com)

**This file includes:**

**Supplementary Table S1-S3.**

**Table S1.** Inter- and intra-day precision of Lx2-32c in plasma

| Concentration (μg/ml) | Inter-day (RSD %) | Intra-day (RSD %) |
| --- | --- | --- |
| 0.1 | 1.4 | 3 |
| 5 | 1.9 | 3.2 |
| 200 | 2.6 | 3.2 |

**Table S2.** Loading efficiency of Lx2-32c liposome formulation

| Lx2-32c Liposome Concentration (μg/ml) | Lx2-32c Concentration (μg/ml) | Loading efficiency (%) |
| --- | --- | --- |
| 5 | 0.27 | 5.40 |
| 10 | 0.53 | 5.30 |
| 15  35  50  100 | 0.80  1.61  2.68  5.22 | 5.35  5.36  5.36  5.22 |

**Table S3.** The mean diameter, polydispersity index (PDI) and encapsulation efficiency (EE) of freshly prepared Lx2-32c liposome and surplus formulation after study

|  | Mean diameter (nM) | PDI | EE |
| --- | --- | --- | --- |
| Fresh | 195.5 | 0.114 | 87% |
| Surplus | 225.9 | 0.091 | 83% |
